# Top Factors in Nurses Ending Health Care Employment Between 2018 and 2021

**DOI:** 10.1001/jamanetworkopen.2024.4121

**Published:** 2024-04-09

**Authors:** K. Jane Muir, Joshua Porat-Dahlerbruch, Jacqueline Nikpour, Kathryn Leep-Lazar, Karen B. Lasater

**Affiliations:** 1 Center for Health Outcomes and Policy Research, School of Nursing, University of Pennsylvania, Philadelphia; 2 The Leonard Davis Institute of Health Economics, University of Pennsylvania, Philadelphia; 3 National Clinician Scholars Program, University of Pennsylvania, Philadelphia; 4 Department of Acute and Tertiary Care, School of Nursing, University of Pittsburgh, Pittsburgh, Pennsylvania; 5 Rory Meyers College of Nursing, New York University, New York

## Abstract

**Importance:**

The increase in new registered nurses is expected to outpace retirements, yet health care systems continue to struggle with recruiting and retaining nurses.

**Objective:**

To examine the top contributing factors to nurses ending health care employment between 2018 and 2021 in New York and Illinois.

**Design, Setting, and Participants:**

This cross-sectional study analyzed survey data (RN4CAST-NY/IL) from registered nurses in New York and Illinois from April 13 to June 22, 2021. Differences in contributing factors to ending health care employment are described by nurses’ age, employment status, and prior setting of employment and through exemplar nurse quotes.

**Main Outcomes and Measures:**

Nurses were asked to select all that apply from a list of contributing factors for ending health care employment, and the percentage of nurse respondents per contributing factor were reported.

**Results:**

A total of 7887 nurses (mean [SD] age, 60.1 [12.9] years; 7372 [93%] female) who recently ended health care employment after a mean (SD) of 30.8 (15.1) years of experience were included in the study. Although planned retirement was the leading factor (3047 [39%]), nurses also cited burnout or emotional exhaustion (2039 [26%]), insufficient staffing (1687 [21%]), and family obligations (1456 [18%]) as other top contributing factors. Among retired nurses, 2022 (41%) ended health care employment for reasons other than planned retirement, including burnout or emotional exhaustion (1099 [22%]) and insufficient staffing (888 [18%]). The age distribution of nurses not employed in health care was similar to that of nurses currently employed in health care, suggesting that a demographically similar, already existing supply of nurses could be attracted back into health care employment.

**Conclusions and Relevance:**

In this cross-sectional study, nurses primarily ended health care employment due to systemic features of their employer. Reducing and preventing burnout, improving nurse staffing levels, and supporting nurses’ work-life balance (eg, childcare needs, weekday schedules, and shorter shift lengths) are within the scope of employers and may improve nurse retention.

## Key Points

**Question:**

Why did nurses leave health care employment from 2018 to 2021?

**Findings:**

In this cross-sectional study of 7887 nurses who were employed in a non–health care job, not currently employed, or retired, the top contributing factors for leaving health care employment were planned retirement (39% of nurses), burnout (26%), insufficient staffing (21%), and family obligations (18%). Age distributions of nurses not employed in health care were similar to nurses currently employed in health care.

**Meaning:**

The leading reasons nurses leave health care employment signal opportunities for employers to reattract an existing nurse workforce and retain currently employed nurses.

## Teaser

This cross-sectional study of nurses in New York and Illinois examines the major contributing factors to nurses ending health care employment between 2018 and 2021.

## Introduction

Health care settings, particularly hospitals, are struggling to recruit and retain enough registered nurses (RNs) to fill vacancies, raising concern that the US may be headed toward a national nursing shortage. Counter to common belief, the US has never had more actively licensed nurses than it does today (5.6 million),^1^ with record-breaking graduations from nursing schools and a robust workforce forecast on the horizon, including encouraging growth projected to outpace retirements during the coming decade.^2^ In short, researchers increasingly agree that the current nursing care crisis will not be resolved by producing more nurses because the US already has a healthy supply. Instead, solutions to the care crisis need to address the central reasons why health care employers are failing to attract and retain the current supply of nurses.^3^

Postpandemic evidence from a national sample of nurses working in hospitals known to be good places to work (ie, magnet hospitals) in 2021 shows that 40% of nurses intend to leave their employer in the next year and that the average nurse turnover rate in hospitals is approximately 17%.^4^ Other recent evidence points to long-term understaffing and poor work environments being associated with greater intentions to leave employment^4,5,6,7^; however, less is known about the major factors that cause nurses to ultimately end health care employment.

In this study, we describe the major contributing factors to nurses ending health care employment between 2018 and 2021 and whether those reasons differed by nurses’ age and prior employment setting (eg, hospital and nursing home). We report these factors for leaving health care employment among nurses who are employed but not in health care, not currently employed, and retired.

## Methods

### Study Design

This cross-sectional study was a secondary analysis of survey data from RNs actively licensed in New York and Illinois from April 13 to June 22, 2021. The RN4CAST-NY/IL study^6^ surveyed 100% of RNs in the 2 states via emails derived from state board nursing licensure lists. The survey response rate was 14%, which is within the usual range of response rates for electronic surveys. The primary purpose of the survey was to evaluate nurse demographics, nurse job outcomes, and employment conditions among currently employed nurses. Nurses were asked about their current employment status and were required to select 1 of the following options: employed in health care, employed but not in health care, not currently employed, or retired. Of the 70 072 nurse survey respondents, 15 797 respondents reported being employed but not in health care (n = 2120), not currently employed (n = 3656), or retired (n = 10 021). From these 15 797 respondents, we excluded nurse respondents with missing data on age (n = 220) or their previous setting of employment (n = 9). Nurse respondents reported how long ago they ended their employment in health care (categories: 0-3 months, 4-12 months, 1-3 years, 3-7 years, or ≥7 years), and we further excluded 7681 respondents who ended their employment more than 3 years since the time of the survey to ensure the sample was mostly representative of nurses who left health care employment proximal to the COVID-19 pandemic. Nurses selected from a list of race and ethnicity categories in the survey to inform demographic characteristics of nurses ending health care employment in the study. The final analytic sample included 7887 RNs who left a position in health care employment between April 2018 and June 2021. Participants provided written informed consent before initiating the survey. This study was approved by the University of Pennsylvania Institutional Review Board. The Strengthening the Reporting of Observational Studies in Epidemiology (STROBE) reporting guideline was followed.

### Measures

#### Major Contributing Factors in Ending Health Care Employment

Nurses were asked to select all that apply from a list of contributing factors (eTable 1 in Supplement 1) for ending health care employment, derived from prior published surveys evaluating reasons for nurses’ professional turnover.^8^ Nurses who indicated “other” were asked to write in their reasons, using open-text response. Most (>75%) of the write-in responses were similar to the existing factors on the list and thus were recoded by study coauthors. Three factors from the original list were expanded to incorporate related responses from the write-ins: “Better wages/benefits in other industries” was expanded to “Better benefits, wages, work flexibility in other industries”; “Physical injury” was expanded to “Disability/health status”; and “Employment terminated by employer” was expanded to “Laid off/terminated by employer.” One new category, “Relocation/move,” was created from the write-in responses.

#### Nurse Age, Previous Employment Setting, and Other Factors

Nurses reported their age, which was recoded into categories for analysis (<30, 31-40, 41-50, 51-60, 61-70, and ≥71 years). Nurses also reported the employment setting where they most recently worked from the following list: hospital, nursing home, home care, primary care, or other. If respondents selected “other,” they were asked to write in their setting of previous employment. The final employment categories were hospital, primary and ambulatory care, residential and nursing home, home care and hospice, and other settings.

The final question in the survey was an open-ended text question: “Is there anything else you would like to share about your career as a nurse?” Excluding missing responses, 1325 responses were retained for analysis.

### Statistical Analysis

We reported the number of nurse respondents in our sample overall and by employment status (ie, employed but not in health care, not currently employed, or retired), previous employment setting, and age category. We showed the contributing factors for nurses leaving health care as the percentage of responses across the categories. The percentage of nurses across age categories and employment status were displayed in a density plot and included nurses currently employed in health care for comparison with those nurses employed but not in health care, not currently employed, and retired. Stata software, version 17 (StataCorp LLC) was used for data analysis.

We reviewed open-ended text responses to identify exemplar quotations that further contextualized the quantitative findings.^9,10^ We identified quotations inductively, informed by the quantitative findings of major contributing factors (other than planned retirement) for nurses ending health care employment.^11^

## Results

The sample included 7887 registered nurses who were employed but not in health care (n = 694), not currently employed (n = 2287), or retired (n = 4906). Nurses had a mean (SD) age of 60.1 (12.9) years and 30.8 (15.1) years of experience; 7372 (93%) were female and 515 (7%) were male; and 679 (9%) were Asian, 641 (8%) were Black or African American, 6147 (78%) were White, and 373 (5%) were multiracial or other (American Indian or Alaska Native, Native Hawaiian or Other Pacific Islander, or other) (see additional demographic details in eTable 2 in Supplement 1). The major contributing factors for nurses ending health care employment between April 2018 and June 2021 were planned retirement (3047 [39%]), burnout or emotional exhaustion (2039 [26%]), and insufficient staffing (1687 [21%]) (Table 1). Notably, only 2884 retired nurses (59%) indicated a planned retirement as the contributing factor for ending health care employment, suggesting that 2022 retired nurses (41%) entered retirement unplanned.

**Table 1.** Major Contributing Factors in Nurses Ending Employment in Health Care by Current Employment Status^a^

Factor	No. (%) of nurses			
	All nurses (N = 7887)	Current employment status		
		Employed but not in health care (n = 694)	Not currently employed (n = 2287)	Retired (n = 4906)
Planned retirement	3047 (39)	51 (7)	112 (5)	2884 (59)
Burnout or emotional exhaustion	2039 (26)	283 (41)	657 (29)	1099 (22)
Insufficient staffing	1687 (21)	221 (32)	578 (25)	888 (18)
Family obligations	1456 (18)	126 (18)	724 (32)	606 (12)
Concerns related to COVID-19	1368 (17)	80 (12)	564 (25)	724 (15)
Unsafe working conditions	1047 (13)	136 (20)	425 (19)	486 (10)
Disability or health status	900 (11)	49 (7)	338 (15)	513 (10)
Workplace bullying or violence from colleagues	760 (10)	99 (14)	301 (13)	360 (7)
Other reasons	634 (8)	124 (18)	298 (13)	212 (4)
Not enough opportunity for professional growth and advancement	589 (7)	150 (22)	269 (12)	170 (3)
Better benefits, wages, or work flexibility in other industries	531(7)	193 (28)	190 (8)	148 (3)
Laid off or terminated by employer	568 (7)	36 (5)	307 (13)	225 (5)
Workplace bullying or violence from patients or families	286 (4)	45 (6)	104 (5)	137 (3)
Relocation or move	110 (1)	14 (2)	77 (3)	19 (0.4)

^a^ Respondents could select multiple contributing factors; thus, percentages may not total 100%.

Among retired nurses, other leading factors in their retirement decisions were burnout (1099 [22%]) and insufficient staffing (888 [18%]). Nurses who were employed but not in a health care setting (n = 694) cited burnout or emotional exhaustion (283 [41%]); insufficient staffing (221 [32%]); better benefits, wages, and work flexibility in other industries (193 [28%]); not enough opportunity for professional growth and advancement (150 [22%]); and unsafe working conditions (136 [20%]) as top factors. Among nurses who identified as not currently employed (n = 2287), family obligations (724 [32%]) was the highest ranked factor. A former hospital RN aged 30 to 40 years stated, “I am itching to return to the work force. One thing that has dampened my efforts is childcare available that works with the shifts offered. Another drawback is working every other weekend.”

A total of 3954 nurses (50%) who recently left health care employment previously worked in a hospital (Table 2). Excluding planned retirements, the top contributing factors for ending employment among former hospital nurses were burnout or emotional exhaustion (1128 [29%]), insufficient staffing (964 [24%]), family obligations (721 [18%]), concerns related to COVID-19 (661 [17%]), and unsafe working conditions (644 [16%]). Former residential and nursing home nurses similarly rated burnout or emotional exhaustion (209 [32%]), insufficient staffing (245 [37%]), concerns related to COVID-19 (164 [25%]), unsafe working conditions (157 [24%]), and family obligations (150 [23%]) as their top factors.

**Table 2.** Major Contributing Factors in Nurses Ending Employment in Health Care by Previous Employment Setting^a^

Factor	No. (%) of nurses by previous setting of employment				
	Hospital (n = 3954)	Primary and ambulatory care (n = 1013)	Residential and nursing home (n = 660)	Home care and hospice (n = 697)	Other settings (n = 1563)
Planned retirement	1533 (39)	266 (26)	146 (22)	217 (31)	885 (57)
Burnout or emotional exhaustion	1128 (29)	255 (25)	209 (32)	178 (26)	269 (17)
Insufficient staffing	964 (24)	176 (17)	245 (37)	136 (20)	166 (11)
Family obligations	721 (18)	224 (22)	150 (23)	139 (20)	222 (14)
Unsafe working conditions	644 (16)	90 (9)	157 (24)	68 (10)	88 (6)
Concerns related to COVID-19	661 (17)	189 (19)	164 (25)	149 (21)	205 (13)
Disability or health status	463 (12)	116 (11)	90 (14)	98 (14)	133 (9)
Workplace bullying or violence from colleagues	417 (11)	107 (11)	68 (10)	55 (8)	113 (7)
Other reasons	327 (8)	84 (8)	44 (7)	61 (9)	118 (8)
Not enough opportunity for professional growth and advancement	280 (7)	65 (10)	102 (10)	56 (8)	86 (6)
Laid off or terminated by employer	237 (6)	120 (12)	50 (8)	52 (7)	109 (7)
Better benefits, wages, or work flexibility in other industries	271 (7)	81 (8)	55 (8)	48 (7)	76 (5)
Workplace bullying or violence from patients/families	193 (5)	19 (2)	27 (4)	15 (2)	32 (2)
Relocation or move	48 (1)	21 (2)	NR	NR	21 (1)

Abbreviation: NR, not reported (numbers suppressed due to small cell size).

^a^ Respondents could select multiple contributing factors; thus, percentages may not total 100%. Nurses employed in other settings worked in varied settings, including school nursing, public or community health, academia, and other non–direct care industry jobs.

Nurses described relationships between insufficient staffing, unsafe working conditions, and better benefits or wages as it pertained to their work as a nurse. A former hospital RN aged 60 to 70 years stated, “I would have worked another year or two if we had safe staffing ratios.” A former hospital RN aged 40 to 50 years said, “Unsafe patient nurse ratio, overworked, low staff most of the time. Low wages and poor benefits. Very stressful career with low wages and little employee benefits.” Another former hospital RN aged 40 to 50 years said, “They are struggling to recruit and keep staff because of the conditions versus compensation issue. Patients are sicker and more complex than ever…I was constantly voicing concerns for patient safety because of frontline working conditions…Can I get another nursing job? Absolutely…Do I want to? Not really.” A former hospital RN aged 30 to 40 years stated, “Added expectations, precautions...no transparency in the administration, terrible staffing ratios, risk of giving your family COVID. Weekend and overtime hours, emotional exhaustion. All of this with the same pay and rarely ever any incentives.”

 Factors in ending health care employment varied according to the nurses’ age (Table 3). Among nurses 61 years or older, the highest cited contributing factor was a planned retirement (2308 [53%] of those aged 61-70 years and 587 [55%] in those aged ≥71 years), followed by burnout or emotional exhaustion (980 [23%] of those aged 61-70 years and 107 [10%] in those aged ≥71 years). Among nurses 30 years or younger, burnout or emotional exhaustion (145 [43%]) and insufficient staffing (135 [40%]) were the top factors and were considerably more salient for younger nurses compared with nurses from older age cohorts. It is unclear whether these differences are driven by a survival bias or potentially confounding factors, such as employment setting and position. Among nurses as young as 41 to 50 years, 79 (16%) cited disability or health status as a reason for leaving. Family obligations was the highest cited factor among nurses aged 31 to 40 years (328 [46%]) and was also high among nurses aged 41 to 50 years (182 [37%]). Nurses explained how their work environments affected their decision to leave health care employment. As a former hospital RN younger than 30 years said, “I did not want to leave my team, peers, and patients, but the unsupported weight created by the hospital system was too much to [bear] any longer. In trying to help others become the best version of themselves, I was becoming the worst of mine. I have not ventured back into the healthcare world. I have contemplated leaving the profession altogether.” Another former hospital RN younger than 30 years stated, “The horizontal violence and bullying was obscene. Nurses are still ‘eating their young’ and there is a lot of poor leadership and workplace prejudice against minorities. It’s 2021 and we are still suffering to create a positive work environment. It’s so sad and unimpressive as a young new graduate nurse.” Finally, a former hospital RN aged 60 to 70 years said, “I love working as a nurse. As I got older I found out that the 12 hour shifts were too much on my body. I would still be working if I had the option to work an 8 hour day.”

**Table 3.** Major Contributing Factors in Nurses Ending Employment in Health Care by Age Category^a^

Factor	No. (%) of nurses by age categories, y					
	≤30 (n = 341)	31-40 (n = 714)	41-50 (n = 498)	51-60 (n = 971)	61-70 (n = 4317)	≥71 (n = 1046)
Planned retirement	NR	NR	NR	146 (15)	2308 (53)	578 (55)
Burnout or emotional exhaustion	145 (43)	265 (37)	187 (38)	355 (37)	980 (23)	107 (10)
Insufficient staffing	135 (40)	206 (29)	154 (31)	280 (29)	809 (19)	103 (10)
Family obligations	98 (28)	328 (46)	182 (37)	195 (20)	556 (13)	99 (10)
Unsafe working conditions	89 (26)	122 (17)	104 (21)	202 (21)	471 (11)	59 (6)
Better benefits, wages, or work flexibility in other industries	76 (22)	128 (18)	64 (13)	81 (8)	139 (3)	43 (4)
Not enough opportunity for professional growth and advancement	74 (22)	121 (17)	90 (18)	108 (11)	157 (4)	39 (4)
Concerns related to COVID-19	70 (21)	163 (23)	98 (20)	175 (18)	679 (16)	183 (18)
Other reasons	66 (19)	100 (14)	77 (15)	95 (10)	229 (5)	67 (6)
Workplace bullying or violence from colleagues	45 (13)	85 (12)	74 (15)	158 (16)	351 (8)	47 (5)
Workplace bullying or violence from patients or families	33 (10)	33 (5)	22(4)	55(6)	136 (3)	NR
Disability or health status	29 (9)	59 (8)	79 (16)	193 (20)	461 (11)	79 (8)
Laid off or terminated by employer	11 (3)	40 (6)	45 (9)	98 (10)	296 (7)	78 (7)
Relocation or move	17 (5)	31 (4)	NR	13 (1)	30 (1)	NR

Abbreviation: NR, not reported (numbers suppressed due to small cell size).

^a^ Respondents could select multiple contributing factors; thus, percentages may not total 100%.

The Figure displays the distribution of nurses’ ages by their employment status. Most retired nurses were between the ages of 50 and 80 years, which is consistent with expected patterns of retirement. The age distribution of nurses who are not currently employed or employed in a setting other than health care was similar to the age distribution of nurses employed in health care. Patterns are similar by the distribution of nurses’ years of experience as an RN because age and years of experience are highly correlated (eFigure in Supplement 1).

**Figure.**
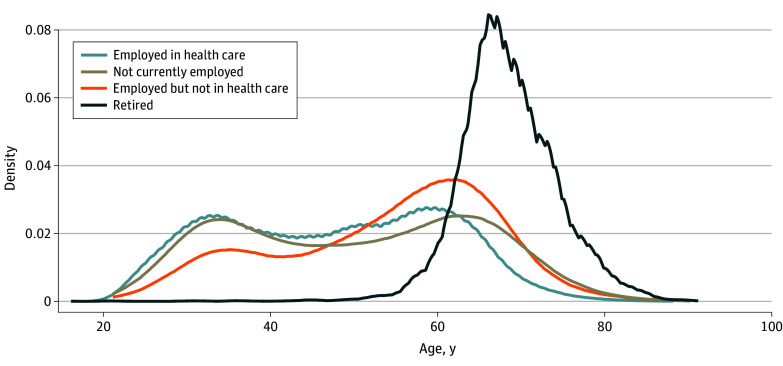
Distribution of Nurses' Age by Employment Status

## Discussion

Besides planned retirement, the leading reasons nurses left health care employment between 2018 and 2021 were burnout or emotional exhaustion, insufficient staffing, family obligations, concerns related to COVID-19, and unsafe working conditions. All of these factors are addressable by the health care employer, including family obligations, which as our first quotation characterizes is a conflict between family caregiving responsibilities and rigid work schedule requirements. That nurses are ending employment because of family obligations suggests that there may be inflexibility on behalf of the employer to support nurses’ continued professional work while also meeting life demands outside the workplace. Indeed, in a 2021 survey of clinicians in magnet hospitals, 32% of physicians and 18% of nurses reported that their work does not allow for personal or family life, suggesting an opportunity for hospitals to improve the work-life balance of clinicians.^4^

We found that 41% of retired nurses left health care employment for reasons other than a planned retirement (with burnout and insufficient staffing as the leading factors), suggesting that what also drives nurses away from health care employment is employers’ challenges providing safe conditions and flexible employment options. This finding is important in the context of concerns over the aging workforce and nursing supply projections. Overall, our findings demonstrate that nonretired nurses largely left health care employment due to issues with their employer rather than personal reasons (eg, going back to school), and more than one-third of retired nurses (41%) experienced an unplanned, premature retirement due to systems issues within their employer.

We also found that the ages (and years of experience) of nurses who are not working in health care but are not yet retired closely resemble those of nurses who are still working in health care. This finding suggests that there is a demographically similar, already existing supply of experienced and actively licensed nurses that could be attracted back into nursing employment. That the nursing care crisis is not driven by a lack of interest in nursing as a career^1^ but rather by a leaky bucket of an already trained, highly experienced nurse workforce is an important finding from our study.^2^

Greater accountability on the part of employers is necessary to address the factors that nurses say drive them away from health care employment—primarily, burnout, insufficient staffing, and family obligations. On the basis of these top contributing factors, employers should invest in safe nurse staffing policies, high-quality nurse work environments, and enhanced opportunities for nurses’ work-life balance and shift work flexibility. Particularly in hospitals and nursing homes,^7,12^ long-term nurse understaffing and high burnout are long-standing organizational issues that predated the COVID-19 pandemic.^5,6,7^ Studies from the US and the UK also find that most nurses who left the profession after 2020 do not cite the pandemic as a major contributor, suggesting that nurse retention problems do not stem from the unique exigencies of the pandemic but rather long-term problems in the workplace, including poor staffing and work environments.^13,14^

In a 2021 survey asking clinicians what interventions would improve their well-being and burnout, both physicians (45%) and nurses (87%) rated “improve nurse staffing levels” as the most important intervention.^4^ More than 2 decades of evidence, including data generated during the COVID-19 pandemic,^4,5,6,15,16,17,18^ consistently finds that the most promising solution, endorsed by physicians and nurses, to reducing clinician burnout and ensuring high-quality, safe patient care is to ensure safe nurse staffing ratios.

Clinician burnout is widely understood to be an occupational phenomenon rather than a factor of individual resiliency or lack thereof.^19,20^ In fact, other studies demonstrate that clinicians are among the most resilient individuals^21^ and give low importance to employer resiliency interventions.^4^ In addition to improving nurses’ workloads, employers can foster better work environments, since the benefits of improving nurse staffing levels are most pronounced in hospitals with good work environments.^22^ Nurses in the current study cited burnout as a primary contributor to leaving health care employment, and numerous other studies have linked better nurse work environments to lower nurse burnout.^23,24,25,26^ Favorable nurse work environments include those with authentic engagement of frontline nurses in organizational decision-making and affairs, including effective and visible nurse leadership in the highest levels of the organization. The Magnet Recognition Program offers an evidence-based blueprint^27^ for structured improvements in nurse work environment features (eg, transformational leadership, structural empowerment, and empirical outcomes) and is associated with better patient (fewer deaths) and nurse (burnout, job dissatisfaction, and intent to leave) outcomes.^23,28,29^

A large proportion of nurses aged 30 to 50 years cited leaving health care employment for family obligations, consistent with other literature that identifies difficulties in work-life balance to be most prevalent for this age group.^30^ Employers could offer more flexible scheduling, on-site childcare, and more generous family leave policies and use market-based incentives, including higher pay differentials than are typically offered for weekends or holiday shifts, vs rotating schedules.^28,29,30,31^ Indeed, evidence exists suggesting that longer shift lengths are associated with 2.5 times higher nurse burnout and job dissatisfaction.^31,32^

Among older nurses with more working experience, disability or health status was a major contributing factor for ending health care employment. Even among nurses as young as 41 to 50 years, 79 (16%) cited disability or health status as a reason for leaving. The physical demands of bedside nursing, including work-related injuries (more likely to occur under conditions of understaffing),^33,34^ may be responsible for preventable workforce exits.^35^ Recent evidence demonstrates that 46% and 69% of nurses of all ages report their health and sleep quality as poor or fair, respectively.^4^ Rigid employer policies that require nurses rotate time of day shifts, weekends, and holidays not only negatively impact nurses’ health but also reduce nurses’ ability to achieve a reasonable work-life balance. Our evidence and other evidence^36,37^ suggest that experienced nurses are motivated to remain in the profession if their employers can reenvision ways that these experienced nurses can contribute their expertise under more flexible employment options.^27^

State and federal policy action can be leveraged to motivate employer accountability in enacting evidence-based solutions likely to retain nurses in health care, such as the Centers for Medicare & Medicaid Services (CMS) Compare platform.^38^ Transparent reporting of hospital nurse turnover and vacancies holds hospitals publicly accountable to establish more favorable work environments that prevent nurse burnout and job dissatisfaction^15,39,40^ and reduce turnover.

Our findings underscore the importance of safe nurse staffing standards as a mechanism for retaining nurses in health care employment. Because many hospitals have failed to voluntarily improve their nurse staffing levels, increasingly more hospitals are facing legislative mandates requiring hospitals to meet minimum safe standards. Although only California and Oregon have mandated minimum nurse staffing requirements in hospitals,^18,41,42^ many other states have pending legislation, including Pennsylvania, Maine, Massachusetts, Michigan, and others. Most recently, an executive of a leading health care system publicly supported the nurse staffing legislation in their state^43^—an example of health care leadership modernizing their approach to respond to the concerns of nurses. Recently, the CMS announced proposed improvements in minimum nurse staffing standards for nursing homes to participate in Medicare and Medicaid; the CMS could potentially take the same approach to set safe nurse staffing standards in hospitals as a condition of participation.^44,45^

### Limitations

This study has some limitations. The provided reasons why nurses ended health care employment are not exhaustive and are potentially correlated (eg, staffing and unsafe working conditions); however, they are informed by prior large empirical surveys^46^ of nurses that similarly ask why nurses left their workplace. We only have a rough estimate of when nurses ended health care employment and therefore cannot confirm whether they left before or during the COVID-19 pandemic. Finally, we only surveyed nurses with active licenses and therefore do not have information for nurses who recently left health care employment and ceased being actively licensed. Although this may present a selection bias, we anticipate the potential bias to be small given that many nurses maintain their license despite entering and exiting health care employment and that most state licenses operate on a 2-year renewal period.

## Conclusions

This cross-sectional study found that, excluding planned retirements, nurses’ major contributing factors to ending health care employment between 2018 and 2021 were (1) burnout and emotional exhaustion, (2) insufficient staffing, and (3) family obligations. These findings are useful to inform policy- and organizational-level solutions for recruiting and retaining nurses in health care. Health care employers’ nurse recruitment and retention problems could be resolved by investing in policies (eg, safe nurse staffing policies) that target the top reasons experienced nurses are leaving (eg, burnout and insufficient staffing) and in flexible opportunities (eg, childcare offerings and flexible shift lengths) that support nurses’ work-life balance.

## Notes

Supplement 1. **eTable 1.** Survey Questions for Why Nurses Ended Healthcare Employment From the RN4CAST-NY/IL Study **eTable 2.** Participant Demographics **eFigure.** Distributions of Nurses’ Years of Experience by Employment Status

Supplement 2.
Data Sharing Statement
