# Supplementary material for: Top Factors in Nurses Ending Health Care Employment Between 2018 and 2021
Source: JAMA Netw Open. 2024 Apr 9;7(4):e244121. doi: 10.1001/jamanetworkopen.2024.4121 (PMC11004833; doi:10.1001/jamanetworkopen.2024.4121)
Supplement: Supplement 1. — eTable 1. Survey Questions for Why Nurses Ended Healthcare Employment From the RN4CAST-NY/IL Study eTable 2. Participant Demographics eFigure. Distributions of Nurses’ Years of Experience by Employment Status [file jamanetwopen-e244121-s001.pdf]

Supplementary Online Content

Muir KJ, Porat-Dahlerbruch J, Nikpour J, Leep-Lazar K, Lasater KB. Top factors in nurses ending health care employment between 2018 and 2021. *JAMA Netw Open*. 2024;7(4):e244121. doi:10.1001/jamanetworkopen.2024.4121

eTable 1. Survey Questions for Why Nurses Ended Healthcare Employment From the RN4CAST-NY/IL Study
eTable 2. Participant Demographics
eFigure. Distributions of Nurses' Years of Experience by Employment Status

This supplementary material has been provided by the authors to give readers additional information about their work.

© 2024 Muir KJ et al. *JAMA Network Open.*

eTable 1. Survey Questions for Why Nurses Ended Healthcare Employment From the RN4CAST-NY/IL Study

**Which best describes your employment status?**
Employed in healthcare
Employed, but not in healthcare
Not currently employed
Retired
**Have you ever been employed in a healthcare setting?**
Yes
No
**What healthcare setting were you most recently employed in?**
Hospital
Nursing home
Home care
Primary care setting
Other: please explain_____
**How long ago did you end your employment in healthcare?**
0-3 months ago
1-3 months ago
1-3 years ago
3-7 years ago
more than 7 years ago
**What were the major contributing factors in ending your employment in healthcare? (select all that apply)**
Concerns related to COVID
Family obligations
Burnout / emotional exhaustion
Physical injury
Workplace bullying/violence from colleagues
Workplace bullying/violence from patients/families
Insufficient staffing
Unsafe working conditions
Better wages/benefits in other industries
Not enough opportunity for professional growth and advancement
Planned retirement
Employment terminated by employer
Other: please explain_____________

© 2024 Muir KJ et al. *JAMA Network Open.*

eTable 2. Participant Demographics

		Current Employment Status			
**Characteristic**	**All nurses**	**Employed but not in healthcare**	**Not currently employed**	**Retired**	**p-value**
	N=7,887	N=694	N=2,287	N=4,906	
**Age, n (SD)**	60.1 (12.9)	48.9 (13.7)	49.3 (14.7)	66.6 (5.3)	<.001
**Years of Experience, n (SD)**	30.8 (15.1)	19.2 (14.9)	18.9 (15.0)	38.0 (9.7)	0.33
**Sex, n (%)***	7,372 (93.7)	609 (88.1)	2,102 (92.2)	4,661 (95.2)	<.001
Male	482 (6)	79 (11)	172 (8)	231 (5)	
Female	7,372 (94)	609 (88)	2,102 (92)	4,661 (95)	
**Race/ethnicity, n (%)****					<.001
Asian	679 (8.7)	111 (16.1)	399 (17.6)	169 (3.5)	
Black or African American	641 (8.2)	89 (12.9)	237 (10.5)	315 (6.5)	
Multiple or Other	373 (4.8)	46 (6.7)	161 (7.1)	166 (3.4)	
White	6,147 (78.4)	445 (64.4)	1,472 (64.9)	4,230 (86.7)	
**Non-Hispanic, n (%)**	7,557 (96.6)	659 (95.9)	2,149 (94.6)	4,749 (97.7)	<.001

* Options were defined by the investigator for the RN4CAST-NY/IL survey. An “Other” category was included in the survey as a write-in option, however the statistics are excluded to adhere to NIH cell size rules.

**Options were defined by the investigator for the RN4CAST-NY/IL survey. Multiple or Other includes: the “Other” category from the RN4CAST-NY/IL survey as well as American Indian, Alaskan, Native Hawaiian or Other Pacific Islander in order to adhere to NIH cell size rules.

© 2024 Muir KJ et al. *JAMA Network Open.*

eFigure. Distributions of Nurses' Years of Experience by Employment Status

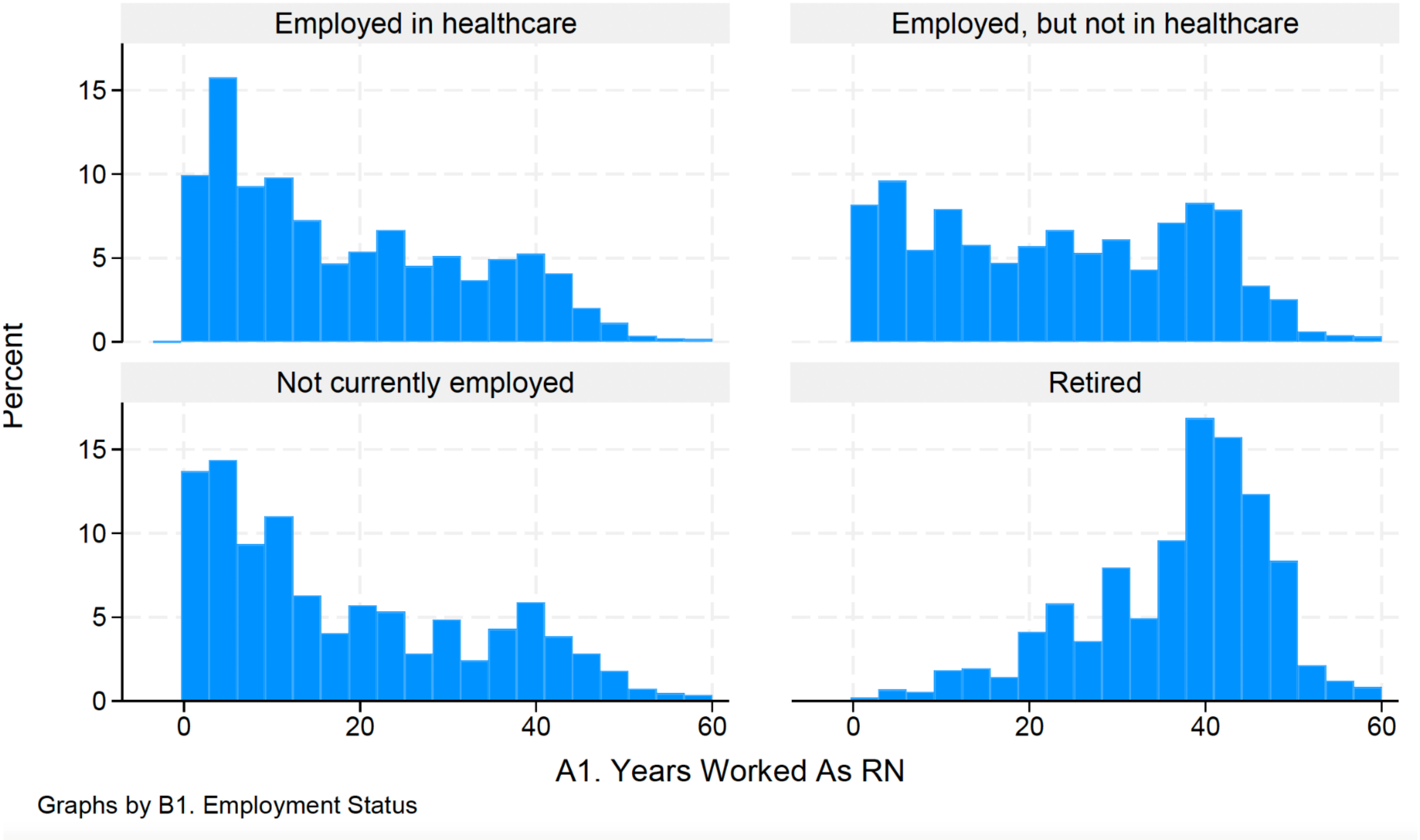

Notes. Distribution of nurses' years of experience among those employed in healthcare (n=54,304), employed but not in healthcare (n=661), not currently employed (n=2,178), and retired (n=4,572). Counts vary from Exhibit 4 due to missingness with nurses' years of experience. Age and years of experience were highly correlated in the study (pwcorr 0.80).

© 2024 Muir KJ et al. *JAMA Network Open.*
