# Supplementary material for: Top Factors in Nurses Ending Health Care Employment Between 2018 and 2021
Source: JAMA Netw Open. 2024 Apr 9;7(4):e244121. doi: 10.1001/jamanetworkopen.2024.4121 (PMC11004833; doi:10.1001/jamanetworkopen.2024.4121)
Supplement: Supplement 2. — Data Sharing Statement [file jamanetwopen-e244121-s002.pdf]

Data Sharing Statement

Muir. Top Factors in Nurses Ending Health Care Employment Between 2018 and 2021. *JAMA Netw Open*. Published April 09, 2024. doi:10.1001/jamanetworkopen.2024.4121

Data
Data available: No

Additional Information
Explanation for why data not available: Data available upon author request.
